# Metabolic regulation and immunosuppressive functions of lipid-associated macrophages in pancreatic ductal adenocarcinoma

**DOI:** 10.3389/fimmu.2026.1865859

**Published:** 2026-06-24

**Authors:** Tongyuan Zhang, Mingze Zhang, Xiaoqin He, Yuefeng Zhang

**Affiliations:** 1 Renmin Hospital of Wuhan University, Wuhan, China; 2 General Administration Office, Renmin Hospital of Wuhan University, Wuhan, China; 3 Hepatopancreatobiliary Surgery, Renmin Hospital of Wuhan University, Wuhan, China

**Keywords:** immunosuppression, lipid metabolism, lipid-associated macrophages, pancreatic ductal adenocarcinoma, tumor immunotherapy, tumor microenvironment

## Abstract

**Introduction:**

Pancreatic ductal adenocarcinoma (PDAC) exhibits a profoundly immunosuppressive tumor microenvironment, with tumor-associated macrophages (TAMs) being the most abundant immune infiltrate. Among them, lipid-associated macrophages (LAMs) have emerged as a distinct subpopulation driving immune evasion through metabolic reprogramming.

**Methods:**

We conducted a narrative review by systematically searching PubMed, Web of Science, and Scopus for articles on LAMs in PDAC up to December 2023. Key themes were synthesized to cover defining markers, metabolic pathways, immunosuppressive functions, and therapeutic strategies.

**Results:**

LAMs are characterized by co-expression of TREM2, APOE, CD9, and lipid-handling genes, and their accumulation correlates with poor prognosis. They undergo metabolic rewiring involving CD36-mediated lipid uptake, dysregulated cholesterol efflux, fatty acid oxidation, and de novo lipogenesis, which collectively enforce an immunosuppressive phenotype. LAMs interact bidirectionally with cancer-associated fibroblasts and directly suppress CD8+ T cells and NK cells. Preclinical targeting of CD36, TREM2, or FAO shows promise but faces challenges in toxicity and delivery.

**Discussion:**

LAMs represent a potential therapeutic vulnerability in PDAC, but the field is still in its early stages. Future work should focus on establishing causal evidence in PDAC-specific models, developing tumor-selective delivery systems, and validating biomarkers for patient stratification.

## 1 Introduction

PDAC accounts for over 90% of pancreatic malignancies and ranks among the most lethal of all digestive system cancers (1). According to the most recent global cancer statistics, PDAC carries a five-year survival rate of approximately 12%, with mortality figures placing it among the leading causes of cancer-related death; projections indicate it will rise to second place by 2030 (2, 3). GLOBOCAN 2020 data report approximately 493,000 new PDAC cases and 460,000 deaths globally (3). The grim prognosis of PDAC stems from multiple clinical challenges: insidious early symptoms, lack of specific diagnostic markers, low surgical resectability rates (approximately 20%), and widespread resistance to chemotherapy and immune checkpoint inhibitors (ICIs). Nonetheless, emerging research on novel immune checkpoint molecules—including LAG-3, TIM-3 and TIGIT—has opened new avenues for overcoming ICI resistance (4, 5).

The pervasive resistance of PDAC to ICIs has drawn intense scrutiny toward its distinctive immune microenvironment (5). Drawing on the classification framework proposed by Teng and colleagues, tumors can be stratified by immune infiltration status into “immune-hot,” “immune-cold” and intermediate phenotypes; PDAC is classified as a canonical “immune-cold” tumor (6). Unlike “immune-hot” tumors such as melanoma and lung cancer, PDAC exhibits a characteristic immune-cold phenotype: tumor-infiltrating lymphocytes (TILs) are sparse, functional CD8+ cytotoxic T cells are severely deficient, while immunosuppressive cells—including regulatory T cells, myeloid-derived suppressor cells and M2-polarized TAMs—are abundantly enriched (7, 8). Among these, TAMs represent the most prevalent immune cell type in the PDAC microenvironment, comprising over 50% of tumor-infiltrating leukocytes, and high-density TAM infiltration correlates significantly with lymph node metastasis, vascular invasion and reduced overall survival (9, 10). The functional heterogeneity of TAMs has been comprehensively described by Binnewies and colleagues (7); Noy and Pollard provided an authoritative synthesis of the multifaceted pro-tumor mechanisms of TAMs in cancer progression (8), while DeNardo and colleagues further delineated the complex relationship between TAMs and cancer immunotherapy (9). The immunosuppressive nature of the PDAC microenvironment integrates multiple layers—including dense stromal fibrosis, diverse immune cell regulatory networks and metabolic reprogramming—and has become a central target for novel therapeutic development (11, 12).

Historically, the M1/M2 polarization framework has been used to interpret the pro-tumor or anti-tumor functions of TAMs; however, this framework fails to capture the true functional heterogeneity of TAMs within the TME (10). The application of single-cell RNA sequencing (scRNA-seq) has enabled systematic dissection of the marked heterogeneity of TAMs (13, 14). In 2019, Jaitin and colleagues first defined LAMs during analysis of adipose tissue from obese mice, describing this macrophage subset as expressing Trem2 (Triggering receptor expressed on myeloid cells 2) and Apoe (Apolipoprotein E) as characteristic lipid-sensing molecules and exerting specific immunoregulatory functions in lipid-laden environments (11). Subsequently, LAMs have been identified across diverse metabolic diseases and tumor microenvironments, including non-alcoholic fatty liver disease, atherosclerosis and obesity-associated cancers (12, 13). Keren-Shaul and colleagues described disease-associated macrophage populations resembling LAMs in mouse models of neurodegenerative disease (12), while Cochain and colleagues revealed the transcriptional heterogeneity of lipid-associated macrophages in atherosclerotic plaques through single-cell sequencing (13); collectively, these cross-disease findings have laid the theoretical foundation for LAMs research. Notably, LAMs were also found to share phenotypic features with tumor-associated macrophages in central nervous system malignancies, underscoring their broad relevance across tissue contexts.

**Figure 1:**
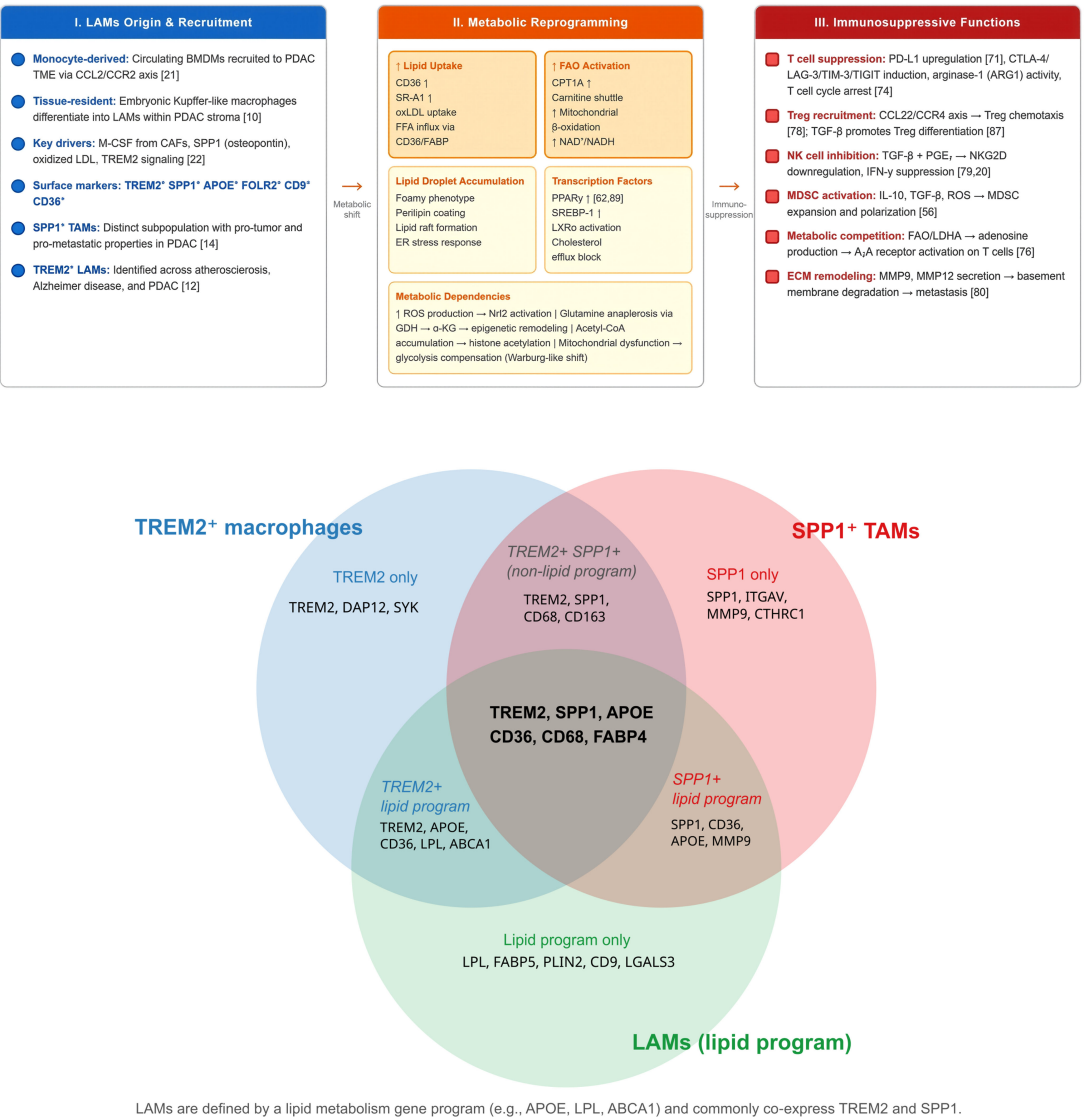
Metabolic reprogramming and immunosuppressive functions of lipid-associated macrophages in PDAC. TME, tumor microenvironment; BMDM, bone marrow–derived monocyte; FAO, fatty acid oxidation; FFAs, free fatty acids; SPP1, osteopontin; TREM2, triggering receptor expressed on myeloid cells 2; APOE, apolipoprotein E; FOLR2, folate receptor beta; CD36, cluster of differentiation 36; CPT1A, carnitine palmitoyltransferase 1A; PPARγ, peroxisome proliferator–activated receptor γ; SREBP-1, sterol regulatory element–binding protein 1; PD-L1, programmed death-ligand 1; TGF-β, transforming growth factor beta; IL-10, interleukin-10; Treg, regulatory T cell; NK, natural killer; MDSC, myeloid-derived suppressor cell; PGE_2_, prostaglandin E2; CTLA-4, cytotoxic T-lymphocyte–associated protein 4; LAG-3, lymphocyte activation gene 3; TIM-3, T-cell immunoglobulin and mucin domain-containing protein 3; TIGIT, T-cell immunoreceptor with Ig and ITIM domains; CCL, C-C motif chemokine ligand (Inset) Venn diagram illustrating the molecular overlap among LAMs, TREM2^+^ macrophages, and SPP1^+^ TAMs.

In the PDAC context, given the lipid-rich nature of the tumor stroma and the central role of metabolic reprogramming in PDAC progression, the functional significance of LAMs has attracted substantial research interest (15, 16). Multiple studies leveraging scRNA-seq datasets from PDAC patient tissues have confirmed that LAMs constitute a numerically substantial and functionally distinct TAMs subpopulation in the PDAC microenvironment, with a deep molecular link between their lipid metabolic activity and immunosuppressive functions (15–17). Recent work by Zhang and colleagues has offered a critical re-examination of the classification of SPP1+ macrophages, providing a new perspective on the taxonomic positioning of LAMs in PDAC (14). This review provides a systematic examination of the multidimensional characteristics of LAMs in PDAC, aiming to serve as a resource for both research advancement and clinical translation in this field (**Figure 1**; **Table 1**).

**Table 1:** LAMs subsets, markers, functions, and therapeutic targets in PDAC.

Category	Surface markers	Immunosuppressive functions	Metabolic/signaling pathways	Therapeutic targets	Ref.
SPP1^+^ TAMs/TREM2^+^ LAMs — PDAC-enriched subsets					
SPP1^+^ TAMs	SPP1^+^ CD68^+^ CD36^+^ APOE^+^	Pro-metastatic; ECM degradation via MMP9; angiogenesis; poor prognosis	SPP1/CD44 signaling; F-κB; IL-6/STAT3	Anti-CCL2 antibodies; CCR2 antagonists; SPP1/CD44 blockade	(14)
TREM2^+^ LAMs	TREM2^+^ CD68^+^ FOLR2^+^ GPR34^+^	Foamy phenotype; lipid-handling; cross-disease presence (cancer, atherosclerosis, neurodegeneration)	TREM2/SYK signaling; PI3K/AKT; lipid raft formation	TREM2 agonists/antagonists; SYK inhibitors; lipid raft disruptors	(12, 18)
FOLR2^+^ LAMs — perivascular niche LAMs					
FOLR2^+^ LAMs	FOLR2^+^ CD163^+^ CD36^+^ MRC1^+^	Perivascular lipid processing; neovascularization support; HIF-1α-driven	HIF-1α under hypoxia; folate metabolism; VEGF co-expression	Anti-FOLR2 conjugates; folate-targeted drug delivery; hypoxia-targeting agents	(18)
Lipid metabolism reprogramming — CD36/PPARγ axis					
CD36^+^ LAMs	CD36^+^ CD68^+^ FABP4/5^+^	Major fatty acid transporter; lipid droplet formation; T cell suppression via metabolic competition	CD36/FABP → FFA influx → CPT1A-mediated FAO → TCA cycle → NAD^+^/NADH shift; Nrf2/ROS axis	CD36 inhibitors (SSO, anti-CD36 mAb); FABP4/5 inhibitors; FAO inhibitors (etomoxir, perhexiline)	(19–21)
PPARγ^+^ LAMs	PPARγ^+^ CD68^+^ ABCA1^+^	Lipid storage regulation; anti-inflammatory gene transcription; foam cell formation	PPARγ-LXRα-ABCA1 pathway; cholesterol efflux blockade; TGF-β/Smad signaling	PPARγ agonists (thiazolidinediones); LXR agonists; ABCA1 modulators	(20, 22)
Immunosuppressive effector functions					
Cytokine producers	CD68^+^ IL-10^+^ TGF-β^+^ ARG1^+^	IL-10: APC inhibition; TGF-β: Treg differentiation; ARG1: L-arginine depletion → T cell anergy	STAT3/STAT6; NF-κB; SMAD; JAK/STAT	Anti-IL-10 antibodies; TGF-β inhibitors (galunisertib); ARG1 inhibitors; STAT3 inhibitors	(23–25)
Checkpoint regulators	PD-L1^+^ CD68^+^ B7-H4^+^	PD-1/PD-L1 direct T cell inhibition; B7-H4 blocks T cell activation; synergy with hypoxia	IFN-γ/JAK/STAT1; HIF-1α stabilization; PTEN loss → PI3K/AKT	Anti-PD-L1/PD-1 ICIs; anti-B7-H4 antibodies; HIF-1α inhibitors; PI3Kγ inhibitors (IPI-549)	(5, 26)
Treg recruiters	CCL22^+^ CD68^+^ CCL17^+^ CCR4^+^	CCL22/CCL17 → CCR4^+^ Treg chemotaxis; local Treg enrichment; immune tolerance amplification	STAT3-mediated CCL22 transcription; TGF-β priming; NF-κB at CCL22 promoter	Anti-CCL22 antibodies; CCR4 antagonists (mogamulizumab)	(27)
NK cell inhibitors	TGF-β^+^ PGE_2_^+^ IL-10^+^	TGF-β + PGE_2_ → NKG2D downregulation on NK cells; IFN-γ suppression; impaired cytotoxicity	COX-2/PGE_2_/EP2-EP4 axis; SMAD-dependent TGF-β; adenosine/A_2_A receptor	COX-2 inhibitors (celecoxib); EP2/EP4 antagonists; TGF-β blockade; A_2_A receptor antagonists	(28, 29)
LAMs–MDSCs–CAFs crosstalk					
CAFs–LAMs–MDSCs axis	iNOS^+^ CD11b^+^ CD33^+^ CD15^+^	CAFs → IL-6/IL-10 → LAMs/MDSCs activation → ROS/RNS → T cell suppression; MMP9 → ECM degradation → metastasis	IL-6/STAT3; ROS/NOS; CXCL12/CXCR4	STAT3 inhibitors (WP1066); CXCR4 antagonists (plerixafor); CSF1R inhibitors; MDSC-depleting agents (ATRA)	(30–32)
Prognostic significance					
Prognostic biomarkers	SPP1^+^ CD68^+^ TREM2^+^ CD36^+^	High SPP1^+^ LAMs → shorter OS; TREM2^+^ LAMs → ICIs resistance; CD36^+^ LAMs → lipid accumulation correlates with grade	SPP1/CD44 → EMT; TREM2 → metabolic rewiring; CD36 → FAO dependence	Predictive biomarker panel for ICIs; combination with gemcitabine + nab-paclitaxel; neoadjuvant stratification	(14, 33, 34)
Therapeutic targeting strategies					
CSF1R axis	CSF1R^+^ CD68^+^	CSF1R blockade → TAMs repolarization M2→M1; reduces LAMs burden; synergistic with PD-1 blockade	CSF1/CSF1R → PI3K/AKT/mTOR; CSF1R → MAPK	CSF1R inhibitors (pexidartinib, BLZ945); anti-CSF1R antibodies	(35, 36)
CD47-SIRPα	CD47^+^ CD68^+^	CD47-SIRPα prevents macrophage phagocytosis; blockade enhances efferocytosis and antigen presentation	SIRPα → SHP-1/SHP-2 → actin reorganization inhibition	Anti-CD47 (magrolimab); SIRPα-Fc (TTI-621); + anti-PD-1 combination	(37)
Lipid metabolism	CD36^+^ FAO^+^	CD36 inhibition → ferroptosis sensitization; FAO blockade → ROS accumulation → LAMs depletion; synergistic with ICIs	CD36 → CPT1A → FAO; ACSL enzymes; ACSL4 → ferroptosis	CD36 inhibitors; CPT1A inhibitors (etomoxir); ACSL4 activators; lipid metabolism + ICI	(21, 38)
Nanoparticle delivery	LAMs targeting (FOLR2, TREM2)	Lipid-based NPs preferentially accumulate in LAMs; folate-conjugated NPs target FOLR2^+^ LAMs; mannose-coated NPs target MRC1^+^ TAMs	Receptor-mediated endocytosis; passive EPR + active targeting	FOLR2-targeted NPs; TREM2-targeted liposomes; mannose micelles; gemcitabine combinations	(39, 40)

SPP1, secreted phosphoprotein 1 (osteopontin); TREM2, triggering receptor expressed on myeloid cells 2; FOLR2, folate receptor beta; CD36, fatty acid translocase; PPARγ, peroxisome proliferator-activated receptor gamma; CSF1R, colony-stimulating factor 1 receptor; ARG1, arginase 1; ICIs, immune checkpoint inhibitors; Treg, regulatory T cell; NK, natural killer; MDSCs, myeloid-derived suppressor cells; CAFs, cancer-associated fibroblasts; ECM, extracellular matrix; MMP, matrix metalloproteinase; FAO, fatty acid oxidation; FFA, free fatty acid; CPT1A, carnitine palmitoyltransferase 1A; LXR, liver X receptor; ABCA1, ATP-binding cassette transporter A1; FABP, fatty acid-binding protein; Nrf2, nuclear factor erythroid 2–related factor 2; ROS, reactive oxygen species; PGE_2_, prostaglandin E2; COX-2, cyclooxygenase-2; ATRA, all-trans retinoic acid; EMT, epithelial-mesenchymal transition; OS, overall survival; SIRPα, signal regulatory protein alpha.

To ensure a transparent and comprehensive evidence base, the literature search was conducted in a systematic manner. This review is a narrative review, not a systematic review, and therefore does not employ a formal PICOS framework or PRISMA flow diagram. However, to maximize rigor, we searched PubMed, Web of Science, and Scopus from inception to December 2023 using combinations of keywords including “lipid-associated macrophages,” “pancreatic ductal adenocarcinoma,” “tumor-associated macrophages,” and “lipid metabolism.” Two reviewers independently screened titles and abstracts, and full texts were assessed against predefined eligibility criteria. The synthesis is qualitative and thematic, aiming to critically appraise the current state of knowledge while explicitly noting the nature of evidence (correlative vs. causal) and the species origin (human vs. mouse) where possible. This approach addresses concerns regarding methodological transparency and allows readers to evaluate the breadth of literature covered.

## 2 Defining markers and single-cell transcriptomic signatures of LAMs

### 2.1 Core molecular markers of LAMs

LAMs are primarily identified through single-cell transcriptomic analysis, and their core markers distinguishing them from other TAMs subpopulations include (**Figure 1**; **Table 1**): (1) TREM2: an immune receptor expressed on myeloid cells that signals through DAP12 and participates in lipid metabolism, phagocytosis and inflammatory regulation; (2) APOE: responsible for lipid transport and metabolism, markedly upregulated in lipid-accumulating macrophages; (3) CD9: a tetraspanin protein involved in cell adhesion, signal transduction and lipid uptake; (4) LGALS3 (Galectin-3): participates in intercellular adhesion and immune regulation; (5) SPP1 (Osteopontin): promotes tumor invasion and immune evasion (41). In addition, LAMs highly express lipid metabolism-associated genes, including FABP4 (fatty acid binding protein 4), FABP5, CD36 (fatty acid translocase), LPL (lipoprotein lipase) and PLIN2 (perilipin 2), which collectively form the molecular basis for the aberrant lipid uptake and accumulation characteristic of LAMs (28, 42). Flow cytometry analyses have confirmed that TREM2+APOE+ cell populations are significantly expanded among PDAC-infiltrating macrophages and exhibit close morphological correspondence to lipid foam-like cells (43). TREM2, as the most specific marker for LAMs, has been systematically validated across multiple solid tumor types as a marker of immunosuppressive tumor-associated monocytes and macrophages (18).

It is important to acknowledge that the definition of LAMs remains in flux and not yet standardized. The transcriptomic signatures used to identify LAMs vary between studies, and there is known overlap with other macrophage subsets such as SPP1+ TAMs and M2−like macrophages. Therefore, many of the mechanisms reviewed here may broadly apply to lipid−laden tumor−associated macrophages rather than to a rigidly defined LAM population. Readers should bear this caveat in mind when interpreting LAM−specific claims.

### 2.2 LAMs features in PDAC single-cell atlases

Multiple scRNA-seq studies on PDAC patient tumor tissues have systematically characterized the transcriptomic profile of LAMs. Steele and colleagues performed high-resolution single-cell atlas analyses of primary tumors, adjacent pancreatic tissue and metastatic lesions from PDAC patients, identifying within the macrophage compartment a LAMs subpopulation defined by co-expression of TREM2, APOE and CD9; this subpopulation was significantly enriched in tumor tissue and correlated inversely with tumor stage and patient prognosis (15, 16). Peng and colleagues performed scRNA-seq on tumor samples from 24 PDAC patients, systematically revealing intra-tumoral cellular heterogeneity, with the pronounced lipid metabolic signature of macrophages and its association with an immunosuppressive phenotype emerging as a particularly notable finding (17). Lin and colleagues integrated multiple PDAC scRNA-seq datasets, identifying a unique transcriptional program in LAMs characterized by marked activation of PPAR signaling and fatty acid β-oxidation pathways alongside significant downregulation of antigen-presenting functions (MHC class II molecules) (44). Although LAMs and classical M2-polarized TAMs share some functional overlap in immunosuppressive capacity, they are molecularly distinct: M2-polarized TAMs are defined by high expression of CD163, CD206 (MRC1), IL-10 and TGF-β, whereas LAMs are centered on TREM2, APOE and lipid metabolic genes; the former are driven by cytokines (IL-4, IL-13), while the latter are driven by lipid metabolic signals, reflecting fundamentally different regulatory pathways (45, 46). The classical review by Mantovani and colleagues provides a systematic framework for understanding macrophage polarization (45), and the guidelines by Murray and colleagues further standardize nomenclature for polarization states (46). Spatial transcriptomics analyses have further demonstrated that LAMs in PDAC frequently localize to regions enriched in adipocytes and stromal fibrosis, forming tight spatial neighborhoods with tumor cells and cancer-associated fibroblasts (CAFs) (47). The review by Wang and colleagues summarizes advances in the application of single-cell sequencing and spatial transcriptomics to PDAC research (48); Shiau and colleagues applied spatial transcriptomics to systematically analyze PDAC patients undergoing neoadjuvant therapy, identifying treatment-associated microenvironment remodeling patterns and providing important data on the dynamic changes of LAMs in the treatment context (49).

A key nuance concerns the relationship between LAMs and the previously described TREM2+/SPP1+ macrophage populations. Single-cell data indicate that LAMs commonly co-express TREM2 and SPP1, but not all TREM2+ or SPP1+ cells are fully equivalent to LAMs, because LAMs are further defined by a lipid-metabolism gene program (e.g., APOE, LPL, ABCA1). The molecular overlap is schematically illustrated by a Venn diagram included in **Figure 1**.

To facilitate species-specific interpretation, we have summarized conserved and species-specific LAM features in **Table 1**. Conserved markers upregulated across human and mouse datasets include TREM2, SPP1, and APOE, whereas species-restricted expression is observed for chemokines such as mouse-specific Ccl8 and human-specific CXCL9 (see **Table 1** for details). Throughout the text, we explicitly denote whether findings originate from human or mouse systems.

## 3 Origin, recruitment and expansion of LAMs in PDAC

### 3.1 Cellular origins of LAMs

Analogous to other TAMs subpopulations, LAMs in PDAC are principally derived from two cellular reservoirs: circulating CCR2+ classical monocytes, which migrate into tumor tissue and differentiate under the influence of the PDAC chemokine milieu, and pancreatic tissue-resident macrophages—originating from embryonic yolk sac and fetal liver hematopoiesis—which undergo phenotypic reprogramming driven by lipid signals within the tumor microenvironment (50, 51). Elyada and colleagues used cross-species single-cell analysis to systematically delineate the immune cell composition and LAMs transcriptional features in PDAC (50). The developmental differentiation trajectories of monocytes, macrophages and dendritic cells have been established by canonical studies (51). Lineage-tracing experiments by Zhu and colleagues demonstrated that pancreatic tissue-resident macrophages begin transitioning toward a pro-tumor phenotype at the earliest stages of PDAC development, serving as important contributors to the establishment of early immunosuppressive TME (52). As tumors progress, bone marrow-derived monocytes progressively become the dominant source of TAMs, including LAMs (53, 54). Sanford and colleagues found that inflammatory monocyte mobilization correlates with poor patient survival in PDAC, a process driven by PDAC-derived chemokines including CCL2, CCL5 and CXCL12 (53, 54). Chen and colleagues reported that loss of type I collagen in the tumor microenvironment intensifies immunosuppression and accelerates tumor progression (55). Recent studies in metabolic dysfunction-associated steatohepatitis (MASH) models have shown that TREM2 is essential for the pro-fibrotic resolution function of LAMs; in lipid-rich tissue microenvironments, TREM2+ LAMs sense lipid signals to regulate their own survival and effector functions, providing important cross-disease insight into TREM2-driven LAMs differentiation programs (56). PDAC initiation and progression involve Kras mutation-driven transition from early pancreatic intraepithelial neoplasia (PanIN) to invasive carcinoma, with immune cell (including macrophage) recruitment and reprogramming commencing at the earliest stages (57).

Despite these advances, the exact ontogeny of LAMs remains an open question. Current evidence supports both a tissue-resident macrophage origin (with lipid-induced *in situ* reprogramming) and a monocyte-derived origin. Definitive lineage-tracing experiments in PDAC models—for instance, using Ms4a3-CreERT2 to track monocytes and Cx3cr1-CreER to label tissue-resident macrophages—are needed to disentangle these possibilities and map the developmental trajectory of LAMs.

### 3.2 Lipid microenvironment driving LAMs recruitment and differentiation

The PDAC stroma contains abundant adipocyte infiltration and tumor necrosis; tumor cell-autonomous aberrant lipid metabolism combined with CAF-mediated extracellular matrix remodeling creates a microenvironment rich in free fatty acids (FFAs), lysophosphatidic acid (LPA) and cholesterol, among other lipid species (57, 58). Mukherjee and colleagues reported that adipocyte-induced FABP4 expression promotes ovarian cancer cell metastasis, highlighting the important pro-tumor role of FABP4 in lipid-rich microenvironments (58). This lipid-rich milieu promotes LAMs differentiation and lipid accumulation through multiple mechanisms: (1) oxidized low-density lipoprotein (oxLDL) and free cholesterol are extensively internalized via CD36 and SR-A (scavenger receptor A), inducing macrophages to transition toward foam cell-like LAMs; (2) LPA activates downstream signaling through LPAR receptors, promoting upregulation of TREM2 and APOE expression; (3) tumor-derived exosomes carrying lipid molecules can be internalized by neighboring macrophages, driving metabolic reprogramming (59, 60). Moreover, the highly active *de novo* lipogenesis in pancreatic cancer cells leads to substantial secretion of saturated and monounsaturated fatty acids; these exogenous lipid species serve as ligands for PPARγ and LXR (liver X receptor), activating macrophage lipid uptake and storage programs and fundamentally reshaping the metabolic phenotype of infiltrating macrophages, thereby driving their differentiation toward LAMs (61, 62). Tumor-derived exosomes also play pivotal roles in immune remodeling of the pre-metastatic niche, where their carried lipid molecules and metabolic signals can induce macrophages at future metastatic sites to adopt an immunosuppressive LAMs-like phenotype (59).

### 3.3 Dynamic changes of LAMs during PDAC progression

Single-cell atlas studies have revealed dynamic changes in LAMs across different stages of PDAC progression. At the early PanIN stage, tissue-resident macrophages begin showing upregulation of lipid metabolic gene expression; as PanIN progresses to invasive PDAC, bone marrow-derived monocytes infiltrate in large numbers and progressively convert to LAMs; in metastatic lesions (with liver metastasis being most common), the proportion of LAMs increases further, suggesting that LAMs may play a specific role in facilitating tumor invasion and metastasis (63, 64). Hwang and colleagues used single-cell and spatial transcriptomics to analyze PDAC samples before and after neoadjuvant chemotherapy, revealing multicellular dynamic remodeling patterns of the tumor immune microenvironment in response to treatment, with LAMs gene signatures significantly enriched in chemotherapy-resistant patients (61). Studies in PDAC mouse models targeting TREM2 have shown that TREM2 depletion alters the immune landscape of the PDAC microenvironment, providing preclinical evidence for LAMs-targeted therapeutic strategies (62).

## 4 Lipid metabolic reprogramming in LAMs

In the following subsections, we synthesize mechanisms while explicitly distinguishing between correlative evidence and causal demonstrations, and between data obtained directly from PDAC models and inferences drawn from other disease contexts.

### 4.1 Lipid uptake: the central role of CD36 and scavenger receptors

CD36 serves as a key mediator of excessive extracellular lipid uptake in LAMs (65). As a multi-ligand scavenger receptor, CD36 binds and internalizes diverse lipid molecules, including oxidized low-density lipoprotein, long-chain fatty acids and phosphatidylserine (66, 67). In the PDAC microenvironment, lipid species released by tumor cells and CAFs are efficiently internalized through CD36, promoting intracellular lipid accumulation in TAMs (66). In PDAC patient tumor samples, the abundance of CD36+ macrophages correlates negatively with overall survival, and *in vitro* experiments have demonstrated that CD36 blockade partially reverses the immunosuppressive functions of LAMs (63). CD36 promotes macrophage uptake of tumor cell-derived extracellular vesicles enriched in long-chain fatty acids, inducing metabolic reprogramming and acquisition of a pro-metastatic phenotype—a mechanism that has been systematically validated in liver metastasis models (68). Chen and colleagues found that the fatty acid receptor CD36 plays a significant regulatory role in tumor-associated immune responses and can promote macrophage infiltration through activation of the p110γ signaling pathway (64, 66). Hale and colleagues first demonstrated the critical role of CD36 in glioma stem cells, confirming that CD36 promotes survival and invasion in tumor-initiating cells (69). Together, these studies indicate that CD36 functions not only as a lipid uptake channel but also as a signaling hub that facilitates LAMs infiltration into tumors and promotes metastasis (66).

However, the majority of studies linking CD36 to LAM functions in PDAC are correlative (e.g., immunohistochemical scoring versus survival) or rely on non-PDAC tumor models. Direct causal validation—for instance, through macrophage-specific Cd36 deletion in autochthonous PDAC mice—is still lacking. Moreover, foundational insights into CD36-mediated lipid uptake originate largely from atherosclerosis research; their direct applicability to the PDAC microenvironment remains to be established.

### 4.2 Cholesterol metabolism dysregulation and immune dysfunction

Cholesterol metabolism dysregulation constitutes a core metabolic feature of LAMs. Cholesterol levels are abnormally elevated in the PDAC microenvironment, derived from both increased tumor cell synthesis (upregulated HMGCR pathway) and exogenous uptake (upregulated LDL receptor) (70, 71). LAMs upregulate cholesterol uptake (LDL receptor, CD36) while downregulating cholesterol efflux (ABCA1, ABCG1), resulting in intracellular free cholesterol accumulation. Excess free cholesterol, beyond potentially forming crystalline deposits that activate the NLRP3 inflammasome, impairs the anti-tumor functions of LAMs through several additional mechanisms: (1) altering cell membrane fluidity and lipid raft composition, affecting receptor signaling and immune synapse formation; (2) suppressing MHC class II molecule expression and impairing antigen-presenting capacity; (3) increasing the production of oxidized sterols such as 25-hydroxycholesterol (35). The reviews by Spann and Glass provide an in-depth account of the critical roles of sterols and oxysterols in immune cell function regulation (35), while Galvan-Pena and O’Neill systematically reviewed metabolic reprogramming during macrophage polarization (19). APOE, highly expressed by LAMs, participates in cholesterol internalization and redistribution through binding to APOE receptors (LRP1 and others), and promotes PDAC immunosuppressive microenvironment through NF-κB-mediated CXCL1 production (43). Tumor APOE has also been identified as a critical immune checkpoint blocking anti-tumor immunity through multiple mechanisms that suppress effector immune cell activation (72).

The mechanistic framework linking macrophage cholesterol overload to immune dysfunction has been built predominantly in atherosclerotic and metabolic disease models. While these concepts provide valuable paradigms, the existence and functional consequences of such cholesterol-dependent immunosuppression specifically in PDAC-infiltrating LAMs require more targeted investigation.

### 4.3 Fatty acid oxidation and immune phenotype remodeling

Fatty acid β-oxidation (FAO) plays a critical role in the metabolic program of LAMs. Unlike M1-polarized macrophages that rely on glycolysis, LAMs preferentially utilize mitochondrial FAO for energy production, and this metabolic preference is tightly coupled to their immunosuppressive phenotype (73, 74). In the PDAC microenvironment, free fatty acids released by tumor cells and adipocytes are taken up by LAMs, activated by acyl-CoA synthetases (ACSLs) and entered into mitochondrial β-oxidation, generating abundant NADH and FADH2 for oxidative phosphorylation (OXPHOS) while producing acetyl-CoA that enters the TCA cycle (30, 75). FAO activation drives the immunosuppressive phenotype of LAMs through multiple mechanisms: FAO-dependent low-level production of mitochondrial reactive oxygen species (mtROS) favors anti-inflammatory gene expression; FAO metabolites activate PPAR signaling pathways, transcriptionally activating immunosuppressive factors such as IL-10 and TGF-β; FAO maintains the NAD+/NADH balance, supporting the activity of sirtuins (SIRT1/SIRT3) and related deacetylases, which in turn negatively regulate pro-inflammatory signaling pathways including NF-κB (76–78). Li and colleagues reviewed the application of targeting ROS and FAO in TAMs therapy (76). Lysosomal lipid hydrolysis is an essential prerequisite for macrophage alternative activation; the landmark study by Huang and colleagues revealed the necessity of intracellular lysosomal lipolysis for macrophage alternative activation, with its products driving the FAO metabolic program and maintaining the immunosuppressive phenotype through the PPARδ signaling axis (31, 70). The review by Lim and colleagues systematically summarized the close relationship between T cell lipid metabolism and effector function—a mechanism highly relevant to the context of LAMs-mediated T cell metabolic competition (20, 71).

At present, the evidence that FAO is the preferential metabolic program of LAMs derives largely from transcriptomic profiling and *in vitro* pharmacological inhibition. *In vivo* flux analyses in PDAC are absent, and many key experiments were performed in other tumor or metabolic disease settings. Thus, the extent to which FAO is truly indispensable for LAM-mediated immunosuppression in PDAC awaits causal *in vivo* validation.

### 4.4 SREBP/FASN-mediated *de novo* lipogenesis

Notably, LAMs do not merely passively take up exogenous lipids; their endogenous *de novo* lipogenesis pathways also exhibit a degree of activation. SREBP-1 (sterol regulatory element binding protein 1) is the master transcription factor regulating fatty acid and sterol synthesis (73). The landmark review by Menendez and Lupu articulated the central role of fatty acid synthase (FASN) in tumor metabolism and lipogenesis (79). Under stimulation by Th2 cytokines such as IL-4, SREBP-1 is substantially upregulated, and this upregulation is further enhanced in the PDAC microenvironment by signals derived from CAFs (74). Bidault and colleagues revealed that SREBP1 influences macrophage inflammatory status through lipid synthesis regulation—SREBP-1 activation drives upregulation of FASN and acetyl-CoA carboxylase (ACC), promoting *de novo* synthesis of fatty acids such as palmitic acid and oleic acid; SREBP-1-induced fatty acid synthesis can also deplete the antioxidant defense capacity of macrophages, pushing them toward an alternative activation phenotype (73). These endogenously synthesized fatty acids serve on one hand as PPARγ ligands that sustain the anti-inflammatory program of LAMs, and on the other hand participate in cell membrane phospholipid remodeling, altering the distribution and function of signaling receptors (74). Park and colleagues explored the potential of PPARγ agonists in reprogramming tumor-associated macrophages (21). Experimental studies with FASN inhibitors have shown that blocking FASN partially restores the M1 pro-inflammatory function of macrophages, suggesting that *de novo* lipogenesis makes an important contribution to maintaining LAMs immunosuppression (30).

Studies on *de novo* lipogenesis in LAMs are still in their infancy, and current evidence is predominantly correlative—based on gene expression signatures rather than functional metabolic assays. The contribution of SREBP-1/FASN to the maintenance of LAM immunosuppression in PDAC requires direct intervention studies.

## 5 Effector mechanisms by which LAMs mediate immunosuppression in PDAC

### 5.1 Impaired antigen presentation and T cell exclusion

Effective anti-tumor immunity depends on professional antigen-presenting cells (APCs) processing and presenting tumor-associated antigens to T cells. LAMs impair this process through multiple mechanisms: (1) lipid accumulation leads to marked downregulation of MHC class II molecule surface expression, suppressing CD4+ helper T cell activation; (2) lysosomal lipid overload interferes with antigen processing and degradation, affecting the formation of antigen peptide-MHC class II complexes; (3) APOE secreted by LAMs directly inhibits T cell receptor (TCR) signaling by binding to LRP receptors on T cell surfaces, attenuating T cell activation and proliferation (16, 22). Furthermore, LAMs highly express PD-L1 (CD274), which directly binds PD-1 on infiltrating T cells to induce T cell functional exhaustion; the large amounts of VEGF-A secreted by LAMs further inhibit normalization of tumor vasculature, obstructing effector T cell migration into the tumor core (80, 81). Systemic dysregulation of type 1 conventional dendritic cells in pancreatic cancer also represents an important contributor to impaired anti-tumor immunity, and together with LAMs constitutes a dual mechanism of antigen presentation deficiency in PDAC (44).

Although such mechanisms have been well-characterized *in vitro* and in other tumor types, their operation specifically within the lipid-rich PDAC microenvironment warrants further confirmatory studies.

### 5.2 Production of immunosuppressive cytokines

LAMs are a major source of IL-10 and TGF-β in the PDAC microenvironment. TGF-β exerts pleiotropic effects in tumor progression, promoting the differentiation of Foxp3+ regulatory T cells (Tregs), inhibiting the cytotoxic activity of natural killer (NK) cells and inducing T cell functional exhaustion—collectively suppressing anti-tumor immune responses at multiple levels (23). The authoritative review by Massague systematically summarized the pleiotropic mechanisms of TGF-β in tumor progression (23). Beyond classical immunosuppressive cytokines, LAMs also produce large quantities of CCL22, which recruits Foxp3+ Treg cells into the tumor microenvironment through CCR4, further reinforcing the immunosuppressive landscape (82). The landmark study by Curiel and colleagues was the first to demonstrate the central role of Treg recruitment through specific chemokine mechanisms in establishing immunosuppressive networks within the tumor microenvironment (82). Recent investigations have revealed that IL-34, a non-canonical CSF1R ligand, can drive tumor-associated macrophage reprogramming to facilitate immune evasion through p53 inactivation; Nian and colleagues found in multiple tumor models that IL-34-mediated macrophage reprogramming leads to lipid metabolic activation and extensive secretion of immunosuppressive cytokines—mechanistic features highly reminiscent of LAMs immunosuppressive pathways in PDAC (83). SPP1 (osteopontin) secreted by LAMs binds to integrins such as ITGAV, activating latent TGF-β precursor cleavage and release, thereby establishing a positive feedback immunosuppressive network; simultaneously, SPP1 promotes macrophage-fibroblast interactions, indirectly exacerbating stromal fibrosis and constructing a physical barrier that further impedes immune cell infiltration (5, 33).

### 5.3 Blockade of macrophage phagocytosis: the CD47-SIRPα axis

“Don’t eat me” signaling represents an important mechanism of tumor immune evasion. PDAC cells highly express CD47, which binds SIRPα on the macrophage surface to transmit inhibitory phagocytic signals (24, 26, 34). Compared with other TAMs subpopulations, LAMs express higher levels of SIRPα and are therefore more sensitive to CD47 signaling, resulting in markedly reduced phagocytic capacity to eliminate tumor cells. Additionally, lysosomal dysfunction caused by intracellular lipid accumulation in LAMs further impairs their ability to degrade phagocytosed material, meaning that even when phagocytic blockade is partially relieved, LAMs struggle to effectively clear tumor cell debris and complete immunogenic antigen presentation (84). The review by Cassetta and colleagues systematically summarized macrophage-targeted therapeutic strategies, providing important context for understanding the therapeutic significance of targeting the CD47-SIRPα axis (85).

### 5.4 Promotion of tumor invasion, angiogenesis and pre-metastatic niche formation

LAMs also play an active role in PDAC invasion and metastasis. LAMs highly secrete matrix metalloproteinases MMP2 and MMP9, degrading extracellular matrix barriers and creating conditions favorable for local tumor cell invasion (77). The review by Kessenbrock and colleagues comprehensively described the central role of MMPs in tumor invasion and metastasis (77). With respect to angiogenesis, LAMs are one of the principal sources of VEGF-A in the PDAC microenvironment; their secreted VEGF-A induces aberrant angiogenesis, forming structurally incomplete tumor vasculature that on one hand facilitates tumor cell entry into the bloodstream and on the other further obstructs transendothelial migration of effector T cells (27, 29). The review by Murdoch and colleagues specifically addressed the regulatory role of myeloid cells in tumor angiogenesis (27). With regard to pre-metastatic niche formation, the landmark study by Costa-Silva and colleagues was the first to systematically demonstrate the critical role of pancreatic cancer exosomes in pre-metastatic niche formation (32); tumor-derived exosomes deliver pro-metastatic signals to the liver, recruiting and activating hepatic Kupffer cells and bone marrow-derived monocytes and converting them to a LAMs-like phenotype (32). Fong and colleagues found that breast cancer-secreted miR-122 reprograms glucose metabolism in the pre-metastatic niche (75), and analogous metabolic reprogramming patterns hold significance in pancreatic cancer liver metastasis (85, 86). Lipid signals carried by tumor-derived exosomes can also induce macrophages in the pre-metastatic niche toward an immunosuppressive phenotype; the study by Morrissey and colleagues systematically elucidated the functional mechanisms of the exosome-macrophage-metastasis axis (59).

Much of the knowledge regarding exosome-mediated pre-metastatic niche formation derives from breast and other cancer models; analogous mechanisms in PDAC liver metastasis are supported by some data but have not yet been fully dissected.

## 6 Interactive networks between LAMs and other microenvironment components

### 6.1 Metabolic crosstalk between LAMs and cancer-associated fibroblasts

CAFs constitute the second-largest cellular compartment after tumor cells in the PDAC microenvironment, secreting large amounts of extracellular matrix proteins that form the characteristic densely fibrotic stroma of PDAC. The systematic review by Sherman and Beatty has elaborated in detail the central role of CAFs in PDAC pathogenesis and therapeutic resistance (80), while the early review by Feig and colleagues laid the groundwork for PDAC microenvironment research (81). There exists complex bidirectional metabolic crosstalk between CAFs and LAMs. On one hand, inflammatory CAFs (iCAFs) secrete IL-6, LIF and other cytokines to activate macrophage STAT3 signaling, promoting LAMs phenotype maintenance; mechanically activated CAFs (myCAFs) produce connective tissue growth factor (CTGF) and TGF-β, further enhancing the lipid uptake capacity of LAMs (22, 74). Biffi and colleagues provided deep insight into the mechanisms by which IL-1-mediated JAK/STAT signaling and TGF-β antagonism shape CAF heterogeneity in PDAC, offering an important molecular framework for understanding the interactions between LAMs and different CAF subtypes (74). Conversely, LAMs secrete SPP1, TGF-β and tumor necrosis factor (TNF) to activate the pro-fibrotic program of CAFs, exacerbating matrix deposition (87, 88). Li and colleagues revealed the synergistic pro-tumorigenic microenvironment network formed between CTHRC1+ fibroblasts and SPP1+ macrophages, a reciprocal interaction that plays a central regulatory role in PDAC stromal remodeling (88). This LAMs-CAFs positive feedback loop is strongly supported by spatial transcriptomics data, with the two cell types frequently forming highly colocalized spatial interaction units, representing one of the most potent immunosuppressive axes in the PDAC microenvironment (47).

Some of the described fibroblast-macrophage crosstalk modules have been primarily dissected in fibrotic disease models beyond cancer; their direct validation in PDAC-specific systems would strengthen disease-relevant conclusions.

### 6.2 Metabolic competition and mutualism between LAMs and tumor cells

Tumor cells and LAMs engage in both metabolic competition and mutualism over substrate utilization. PDAC tumor cells achieve substantial autonomous lipid supply by upregulating key lipid synthesis enzymes (FASN, ACC1, SCD1); simultaneously, they secrete large quantities of lipid-enriched exosomes to “offload” excess lipid burden to neighboring macrophages, inducing their transition toward LAMs, thereby accomplishing metabolic mutualism (23, 84). Reciprocally, LAMs help regulate local lipid concentrations through secretion of fatty acid binding proteins (FABPs) and lipoprotein lipase (LPL), maintaining a favorable metabolic microenvironment for tumor cell survival (89). Moreover, the highly active phagocytosis of LAMs clears cell debris from necrotic tumor areas, preventing large-scale release of damage-associated molecular patterns (DAMPs) from dying tumor cells that would otherwise activate anti-tumor immunity, thereby creating a safer microenvironment for tumor cells (84). Of note, pyrimidine metabolites released by macrophages can directly inhibit the cytotoxic activity of gemcitabine against pancreatic cancer cells, revealing a metabolic mechanism by which TAMs mediate chemotherapy resistance (33).

### 6.3 Direct suppression of CD8+ T cells and NK cells by LAMs

Beyond indirect effects through cytokines, LAMs can directly contact and suppress CD8+ T cells and NK cells. LAMs highly express PD-L1 and other co-inhibitory ligands, which bind their corresponding receptors on T cell surfaces to induce T cell exhaustion (29). At the metabolic level, LAMs deplete microenvironmental arginine through high expression of arginase 1 (ARG1) (87) and consume tryptophan through the IDO pathway, and both of these amino acids are critical metabolic substrates for T cell proliferation and function; their local depletion leads to metabolic restriction and functional impairment of effector T cells (20, 90). The review by Raber and colleagues provided a detailed account of the regulatory role of arginine metabolism in tumor immunity (87). Prostaglandin E2 (PGE2) secreted by LAMs not only directly inhibits NK cell cytotoxic activity but also suppresses T cell IL-2 production and proliferation through EP2/EP4 receptors, constituting a multi-target immunosuppressive network (25). Myeloid-derived suppressor cells (MDSCs) are also important regulatory elements in the PDAC immune microenvironment, and together with LAMs they constitute the myeloid immunosuppression axis (70).

## 7 Therapeutic strategies targeting LAMs

### 7.1 Interfering with LAMs recruitment: CCR2/CCL2 and CXCL12/CXCR4 axis blockade

Blocking monocyte migration to tumor sites is a direct strategy to reduce TAMs (including LAMs) infiltration. The CCL2/CCR2 axis represents the principal chemokine axis mediating monocyte recruitment to PDAC tumors, and multiple preclinical studies have confirmed that CCR2 antagonists significantly reduce intratumoral TAMs infiltration and enhance chemotherapy efficacy (53). The foundational study by Nywening and colleagues systematically validated the therapeutic potential of a dual strategy targeting CXCR2+ neutrophils and CCR2+ macrophages in PDAC (53). A phase I clinical trial of PF-04136309 (a CCR2 antagonist) in combination with FOLFIRINOX showed encouraging early response rates; however, a subsequent phase II study failed to meet its primary endpoint, highlighting the limitations of single-target recruitment blockade (91). The CXCL12/CXCR4 axis also participates in bone marrow-derived cell recruitment in PDAC; BL-8040 (a CXCR4 antagonist) has been evaluated in combination with pembrolizumab in the COMBAT trial, with results suggesting that CXCR4 blockade increases CD8+ T cell infiltration and correlates with LAMs reduction (38, 86).

Despite initial signals of activity, the failure of the subsequent phase II CCR2 trial highlights the challenges of single-target chemokine blockade, including compensatory upregulation of alternative recruitment pathways (e.g., CXCL12/CXCR4) and the potential for systemically suppressing protective monocyte functions. Future strategies may require dual or sequential blockade.

### 7.2 Targeting the CSF1/CSF1R axis to promote LAMs depletion and reprogramming

Colony-stimulating factor 1 receptor (CSF1R) signaling is a critical dependency for the survival, proliferation and differentiation of TAMs (including LAMs), and CSF1R inhibitors can effectively deplete or reprogram intratumoral macrophages (92). Multiple CSF1R inhibitors (including pexidartinib, PLX3397 and IMC-CS4) have been evaluated in PDAC clinical trials, with monotherapy showing limited efficacy; exploratory studies combining these agents with chemotherapy or ICIs have demonstrated some synergistic sensitizing effects (93). Cassetta and Pollard provided a systematic review of macrophage-targeted cancer therapeutic strategies (85). Notably, the effect of CSF1R inhibitors extends beyond LAMs depletion; by clearing immunosuppressive macrophages, these agents can shift the PDAC microenvironment from “cold” to “hot,” laying the groundwork for subsequent immunotherapy (84). Recent research suggests that LAMs may be less sensitive to CSF1R inhibitors than classical M2-polarized TAMs, highlighting the need to develop more precise depletion strategies targeting LAMs-specific markers (62). Macrophage-centric tumor immunotherapy strategies face the dual challenge of depletion versus reprogramming, and their ultimate success will depend on precise identification and intervention against specific subpopulations such as LAMs (94).

Importantly, recent data suggest that LAMs may be less dependent on CSF1R signaling than classical M2 macrophages; thus, CSF1R inhibitors might incompletely deplete this compartment. Systemic toxicities such as hepatotoxicity and fatigue also remain a consideration for clinical translation.

### 7.3 Interfering with lipid metabolism: CD36 inhibition and statin application

Targeting the abnormal lipid metabolism of LAMs has emerged as a rapidly advancing research area. CD36 inhibitors (including SSO, sulfo-N-succinimidyl oleate) have demonstrated in both *in vitro* and *in vivo* models the capacity to reduce tumor foam macrophage formation, restore anti-tumor functions and produce significant synergistic effects with anti-PD-1 therapy (66). Statins inhibit HMGCR to reduce cholesterol synthesis, theoretically alleviating intracellular cholesterol accumulation in LAMs, restoring ABCA1/ABCG1-mediated cholesterol efflux and thereby reversing cholesterol load-associated immunosuppressive functions (71, 72). LXR (liver X receptor) agonists upregulate ABCA1/ABCG1 expression to promote cholesterol efflux, showing some TAMs reprogramming effects in animal models; however, systemic LXR activation-induced hepatic steatosis and other toxicities have limited their clinical application (35, 95). The review by Chen and colleagues specifically discussed the potential of cholesterol efflux as an immunotherapeutic target in tumor-associated macrophages (95). PPARγ agonists have also been explored for blocking lipid metabolism-driven immunosuppressive phenotype in LAMs, with early studies suggesting they can enhance macrophage pro-inflammatory functions (73).

However, systemic CD36 inhibition raises concerns given the receptor’s roles in myocardial fatty acid uptake and platelet function. Similarly, statins have pleiotropic metabolic effects, and LXR agonists can cause hepatic steatosis and hypertriglyceridemia. These potential adverse effects underscore the need for tumor-selective delivery platforms, such as nanoparticle encapsulation or antibody-drug conjugates targeting LAM-specific surface markers.

### 7.4 Targeting TREM2: an emerging strategy for LAMs functional modulation

TREM2, as the most specific marker of LAMs, represents a priority drug target for LAMs-directed therapy. Katzenelenbogen and colleagues demonstrated that TREM2+ tumor-associated macrophages are key mediators of immunosuppression across multiple solid tumor types, and that targeting TREM2 can effectively inhibit LAMs lipid metabolic programs and restore their pro-inflammatory functions (41). Molgora and colleagues found in multiple tumor models that anti-TREM2 antibody therapy drives robust anti-tumor immune responses, significantly reducing intratumoral LAMs proportion and increasing effector T cell infiltration, with synergistic effects when combined with anti-PD-1 therapy (96). The depletion effect of TREM2 in PDAC has been validated in recent studies, which systematically demonstrated the immune microenvironment remodeling induced by TREM2 depletion, providing important translational evidence for clinical targeting (62). Emerging reports on TREM2 targeting include bispecific antibody strategies (simultaneously targeting TREM2 and T cell activation markers) and antibody-drug conjugate (ADC) approaches, aiming to achieve precise LAMs depletion and functional reprogramming (37).

Because TREM2 is also expressed on microglia and other myeloid cells, systemic blockade may carry unintended neurological or metabolic consequences. Restricting TREM2 inhibition to the tumor microenvironment—via conditionally active antibodies or local delivery—would be a prudent avenue.

### 7.5 Metabolic reprogramming combined with immunotherapy

Given that the immunosuppressive functions of LAMs are rooted in their metabolic reprogramming, combination strategies restoring metabolic normalization together with immune activation hold considerable promise (97). Drugs targeting FAO (such as etomoxib, ranolazine and other CPT1A inhibitors) can block the β-oxidation preference of LAMs and promote their transition toward glycolysis-dependent M1 polarization; when combined with ICIs, this metabolic reprogramming effect is substantially amplified (30). The review by Bian and colleagues addressed lipid metabolic reprogramming in TAMs (97), and the review by Zhou and colleagues comprehensively summarized the metabolic reprogramming mechanisms of tumor-associated macrophages (36). Blocking the IL-4/IL-13 signaling pathway—the upstream signal driving LAMs SREBP-1 activation—has shown potential for reprogramming LAMs and enhancing immunotherapy efficacy in multiple tumor models (74). The review by Liu and colleagues systematically summarized multiple drug strategies targeting macrophage mitochondrial metabolism, glycolysis and lipid synthesis pathways (22, 39), and the review by Luo and colleagues summarized the emerging roles of lipid metabolism in tumor-associated macrophages, confirming that metabolic reprogramming can effectively reverse the immunosuppressive phenotype of TAMs and synergize with ICIs—this comprehensive framework provides important theoretical support for metabolic targeting of LAMs in PDAC (98). Additionally, nanoparticle technology offers a precise delivery platform for LAMs-targeted lipid metabolic intervention; several lipid-drug-loaded nanoparticles have demonstrated microenvironment remodeling and enhanced immunotherapy efficacy in PDAC animal models (99, 100). The review by Tanaka and colleagues systematically summarized the role of lipid metabolism in regulating tumor immune cell functions, providing broader theoretical support for the aforementioned metabolic targeting strategies (99).

Pharmacological FAO inhibition can trigger compensatory metabolic rewiring, with macrophages switching to glutamine or glucose metabolism to maintain energy homeostasis. This plasticity may limit the durability of FAO-based monotherapies. Additionally, because cardiac muscle relies heavily on FAO, systemic CPT1A inhibitors have, in some contexts, been associated with myocardial toxicity. Concomitant blockade of compensatory pathways or the use of targeted delivery systems may be necessary to realize the full potential of this approach.

### 7.6 Clinical translation challenges and opportunities

The therapeutic targeting of LAM lipid metabolism holds considerable promise but must confront several overarching translational hurdles. First, most proposed interventions remain at the preclinical stage, and their safety profiles in PDAC patients—who often present with cachexia and metabolic comorbidities—are poorly defined. Second, systemic modulation of lipid pathways inevitably perturbs whole−body homeostasis, potentially causing dyslipidemia, hepatic steatosis, or cardiac impairment. Third, the adaptive nature of cellular metabolism means that LAMs may rapidly engage compensatory pathways, as exemplified by the shift to glutamine metabolism upon FAO inhibition.

To mitigate these risks, strategies such as tumor− or macrophage−specific nanocarriers, intermittent dosing regimens, and simultaneous targeting of complementary metabolic nodes are being explored. Furthermore, biomarker−driven patient selection will be critical to identify those individuals whose tumors are most reliant on LAM−mediated immunosuppression. Rigorous PDAC−specific preclinical models that recapitulate the lipid−rich microenvironment, combined with early−phase clinical trials incorporating pharmacodynamic biomarkers, are essential to translate these exciting concepts into safe and effective therapies.

## 8 Prognostic value and biomarker potential

The association between high LAMs infiltration and poor prognosis in PDAC patients has been validated across multiple independent cohorts. LAMs signature scores constructed from scRNA-seq data can effectively stratify PDAC patients into distinct prognostic subgroups, with high LAMs scores associated with significantly shorter overall survival (OS) and progression-free survival (PFS) (15, 16). Immunohistochemistry analyses show that TREM2+APOE+ macrophage density is an independent predictor of poor PDAC prognosis beyond tumor stage, suggesting its potential utility as a pathological prognostic marker. Moreover, LAMs-associated gene expression features in peripheral blood monocytes and plasma soluble TREM2 (sTREM2) levels are being explored as non-invasive liquid biopsy indicators for predicting immunotherapy response in PDAC patients (25). LAMs infiltration abundance also correlates significantly with resistance to FOLFIRINOX chemotherapy, with high LAMs-infiltrating patients showing notably lower objective response rates (ORR) to first-line chemotherapy, suggesting that LAMs assessment may guide individualized first-line treatment selection (15, 16). The review by Zhang and colleagues systematically summarized the role of lactate in tumors and inflammation, further revealing potential links between metabolic microenvironment and LAMs functions (40). The immunophenotypic classification of intra-tumoral inflammatory status (“hot,” “cold” and intermediate) provides a theoretical framework for integrating LAMs biomarkers into personalized treatment decision-making (84).

## 9 Research limitations and future directions

Despite current research having revealed the important functions of LAMs in PDAC, several scientific questions and translational challenges remain to be urgently addressed.

First, the definition and classification criteria for LAMs have not yet been unified. Different research groups employ varying scRNA-seq analytical pipelines, clustering resolutions and marker combinations, resulting in unclear boundaries between LAMs and other TAMs subpopulations (such as SPP1+ TAMs and FOLR2+ TAMs); standardized LAMs identification protocols based on multi-omics integration are needed.

Second, the functions of LAMs are highly dependent on TME context, and there may be substantial gaps between *in vitro* findings and *in vivo* functions. Organoid co-culture systems that accurately recapitulate the lipid-rich PDAC microenvironment (three-dimensional co-culture models containing LAMs, CAFs and tumor cells) will constitute an important technical platform for deeper investigation of LAMs functions.

Third, most current therapeutic strategies targeting LAMs remain at the preclinical stage, with a lack of clinical trials specifically designed for LAMs in PDAC. Conducting prospective biomarker-driven clinical studies and incorporating LAMs-related indicators into companion diagnostic frameworks represents an urgent need to advance clinical translation (101).

Fourth, the spatial heterogeneity and functional diversity of LAMs have not been fully elucidated. Multi-omics spatial analysis combining spatial transcriptomics and mass cytometry (CyTOF) will help precisely localize functional LAMs niches within tumors and decipher their interaction mechanisms with neighboring cellular components (102).

Fifth, the relationship between LAMs and unique pathological features of PDAC such as neural invasion and lymphangiogenesis remains to be established; research in these intersecting domains may reveal broader functional significance of LAMs in PDAC progression. Additionally, the dynamic responses and functional changes of LAMs in the context of neoadjuvant therapy, targeted therapy and radiotherapy deserve in-depth exploration, and related studies will inform optimization of combination treatment strategies.

Sixth, a substantial portion of the reviewed evidence is correlative in nature, derived from transcriptomic profiling or cross-sectional patient studies. Causal relationships between specific lipid metabolic pathways and LAM-mediated immunosuppression have seldom been tested directly in PDAC-specific, conditional gene-knockout models or adoptive transfer experiments. Establishing such causality is a critical next step for the field.

Finally, we reiterate that the LAM definition remains fluid and that many of the discussed mechanisms may extend to broadly defined lipid-laden TAMs. Cross-disease inferences should be interpreted with caution until validated in PDAC-specific systems. Incorporation of standardized LAM identification protocols and multi-omics integration will be instrumental in addressing these limitations.

## 10 Concluding remarks

LAMs constitute a macrophage subpopulation in the PDAC tumor microenvironment characterized by distinctive lipid metabolic features and immunosuppressive functions. Through multiple metabolic mechanisms—including CD36-mediated excessive lipid uptake, cholesterol metabolism dysregulation, FAO metabolic preference and *de novo* lipogenesis activation—LAMs establish a potent immunosuppressive program that deeply contributes to PDAC immune evasion and therapeutic resistance. The complex interactive networks among LAMs, CAFs, tumor cells and T cells further consolidate the immunosuppressive landscape of the PDAC microenvironment(**Figure 1**).

Multiple landmark studies have provided key reference points for understanding LAMs-related mechanisms. Biancur and colleagues systematically analyzed the compensatory properties of metabolic networks in PDAC, offering an important framework for understanding tumor metabolic flexibility (103). Huynh and colleagues revealed the dual functions of CD36 as a fatty acid transporter and signaling molecule (104), while Pascual and colleagues further demonstrated the critical role of CD36 in metastasis-initiating cells (105), and Yang and colleagues elucidated the CD36-mediated metabolic crosstalk network between tumors and macrophages (106). The landmark study by Pyonteck and colleagues confirmed the central regulatory role of CSF1R signaling in macrophage polarization and tumor progression (107). Anderson and colleagues comprehensively summarized advances in tumor microenvironment research, providing a systematic framework for understanding the functional positioning of LAMs within the TME (108). The review by Guo and colleagues systematically addressed the multifaceted roles of lipid droplets in cancer (109), while Zhou and colleagues provided an in-depth analysis of the mechanisms by which FABP5 regulates macrophage lipid metabolism and inflammatory responses (110).

Combination strategies targeting LAMs lipid metabolic axes (CD36, TREM2, cholesterol efflux pathways) together with immune checkpoint inhibitors represent a promising new direction for overcoming the immunotherapy bottleneck in PDAC (**Table 1**). Future research, propelled by concurrent advances in single-cell multi-omics, spatial transcriptomics and clinical translation studies, must further delineate the multidimensional functional landscape of LAMs in PDAC, thereby laying a solid foundation for developing more precise and effective immunometabolic combination therapies for pancreatic cancer. Zhang and colleagues reviewed therapeutic strategies targeting lipid metabolism in cancer, providing important translational reference for the aforementioned directions (111).

It must be emphasized, however, that the field is still in its early stages, and several caveats warrant caution. The definition of LAMs is evolving, and many of the observations discussed may extend to broader lipid-laden TAM populations. Present evidence remains largely correlative or derived from non-PDAC models, and systemic targeting of lipid pathways carries risks of toxicity and compensatory metabolic rewiring. Moving forward, PDAC-specific causal studies, safe delivery platforms, and biomarker-driven clinical trials will be essential to translate this knowledge into meaningful therapeutic advances.

## Data Availability

The original contributions presented in the study are included in the article/supplementary material. Further inquiries can be directed to the corresponding author.

## Glossary

ABCA1: ATP-binding cassette transporter A1
ABCG1: ATP-binding cassette transporter G1
ACC: Acetyl-CoA carboxylase
ACSL: Acyl-CoA synthetase
APCs: Antigen-presenting cells
APOE: Apolipoprotein E
ARG1: Arginase 1
CAFs: Cancer-associated fibroblasts
CCL2: C-C motif chemokine ligand 2
CCR2: C-C chemokine receptor type 2
CCR4: C-C chemokine receptor type 4
CD36: Fatty acid translocase/Cluster of differentiation 36
CD47: Cluster of differentiation 47
CD163: Cluster of differentiation 163
CD206: Cluster of differentiation 206 (MRC1)
CD274: Cluster of differentiation 274 (PD-L1)
cDC1: Type 1 conventional dendritic cells
CPT1A: Carnitine palmitoyltransferase 1A
CSF1R: Colony-stimulating factor 1 receptor
CTGF: Connective tissue growth factor
CTHRC1: Collagen triple helix repeat containing 1
CyTOF: Mass cytometry
DAMPs: Damage-associated molecular patterns
ECM: Extracellular matrix
EP2/EP4: Prostaglandin E2 receptor 2/4
FABP4: Fatty acid binding protein 4
FABP5: Fatty acid binding protein 5
FASN: Fatty acid synthase
FFAs: Free fatty acids
FOLFIRINOX: Fluorouracil, leucovorin, irinotecan and oxaliplatin
Foxp3: Forkhead box P3
GLUT: Glucose transporter
HMGCR: 3-hydroxy-3-methylglutaryl-CoA reductase
iCAFs: Inflammatory cancer-associated fibroblasts
ICIs: Immune checkpoint inhibitors
IDO: Indoleamine 2,3-dioxygenase
IFN-γ: Interferon gamma
IGF: Insulin-like growth factor
IL-4: Interleukin-4
IL-6: Interleukin-6
IL-10: Interleukin-10
IL-13: Interleukin-13
IL-34: Interleukin-34
ITGAV: Integrin alpha V
LAG-3: Lymphocyte-activation gene 3
LPA: Lysophosphatidic acid
LPL: Lipoprotein lipase
LRP1: Low-density lipoprotein receptor-related protein 1
LXR: Liver X receptor
MASH: Metabolic dysfunction-associated steatohepatitis
MDSCs: Myeloid-derived suppressor cells
MHC II: Major histocompatibility complex class II
MMP2: Matrix metalloproteinase-2
MMP9: Matrix metalloproteinase-9
mtROS: Mitochondrial reactive oxygen species
myCAFs: Mechanically activated cancer-associated fibroblasts
NAD+/NADH: Nicotinamide adenine dinucleotide (oxidized/reduced)
NF-κB: Nuclear factor kappa-light-chain-enhancer of activated B cells
NK cells: Natural killer cells
NLRP3: NOD-, LRR- and pyrin domain-containing protein 3
NRF2: Nuclear factor erythroid 2-related factor 2
ORR: Objective response rate
OS: Overall survival
oxLDL: Oxidized low-density lipoprotein
OXPHOS: Oxidative phosphorylation
PanIN: Pancreatic intraepithelial neoplasia
PD-1: Programmed cell death protein 1
PDAC: Pancreatic ductal adenocarcinoma
PD-L1: Programmed death-ligand 1
PFS: Progression-free survival
PGE2: Prostaglandin E2
PLIN2: Perilipin 2
PPARγ: Peroxisome proliferator-activated receptor gamma
PPARδ: Peroxisome proliferator-activated receptor delta
ROS: Reactive oxygen species
SCD1: Stearoyl-CoA desaturase 1
scRNA-seq: Single-cell RNA sequencing
SIRPα: Signal regulatory protein alpha
SIRT1: Sirtuin 1
SIRT3: Sirtuin 3
SPP1: Secreted phosphoprotein 1 (Osteopontin)
SR-A: Scavenger receptor A
STAT3: Signal transducer and activator of transcription 3
TCA cycle: Tricarboxylic acid cycle
TCR: T cell receptor
TGF-β: Transforming growth factor beta
Th2: T helper 2 cell
TIGIT: T cell immunoreceptor with Ig and ITIM domains
TILs: Tumor-infiltrating lymphocytes
TIM-3: T cell immunoglobulin and mucin domain-containing protein 3
TME: Tumor microenvironment
TNF: Tumor necrosis factor
TREM2: Triggering receptor expressed on myeloid cells 2
Tregs: Regulatory T cells
VEGF-A: Vascular endothelial growth factor A.
